# Risk factors associated with in-hospital falls reported to the Patient Safety Commitee of a teaching hospital

**DOI:** 10.31744/einstein_journal/2019AO4432

**Published:** 2019-02-06

**Authors:** Adriane Kênia Moreira Silva, Dayane Carlos Mota da Costa, Adriano Max Moreira Reis

**Affiliations:** 1 Hospital Risoleta Tolentino Neves, Belo Horizonte, MG, Brazil.; 2 Universidade Federal de Minas Gerais, Belo Horizonte, MG, Brazil.

**Keywords:** Fall, Drug therapy, Near miss, healthcare, Patient safety, Risk factors, Pharmaceutical preparations, Queda, Tratamento farmacológico, Near miss, Segurança do paciente, Fatores de risco, Preparações farmacêuticas

## Abstract

**Objective:**

To investigate the use of fall-risk-increasing drugs among patients with falls reported to the Patient Safety Office of a hospital, and to identify the factors associated with high risk for fall.

**Methods:**

A cross-sectional study, carried out in a teaching hospital. The study population was the universe of fall reports received by the Patient Safety Office. The dependent variable was a high risk for falls. The Medication Fall Risk Score was used to measure fall risk. Descriptive, univariate and multivariate analyses were performed.

**Results:**

Of the 125 fall reports in the study, 38 (30.4%) were in 2014, 26 (20.8%) in 2015, and 61 (48.8%) in 2016. Half of the patients (63; 50.4%) were classified as high fall risk and 74 (59.2%) had two or more risk factors for the event. The most frequently used drug classes were opioids (25%), anxiolytics (19.7%), beta-blockers (9.9%), angiotensin II antagonists (7%) and vascular-selective calcium channel blockers (7%). After the adjusted analysis, the factors associated with falls were amputation (odds ratio: 14.17), female sex (odds ratio: 2.98) and severe pain (odds ratio: 5.47).

**Conclusion:**

Medications are an important contributor to in-hospital falls, and the Medication Fall Risk Score can help identify patients at a high risk for falls.

## RESUMO

**Objetivo:**

Investigar o uso de medicamentos que aumentam o risco de queda entre pacientes que tiveram quedas notificadas ao Núcleo de Segurança do Paciente de um hospital, bem como identificar os fatores associados ao risco elevado de queda.

**Métodos:**

Trata-se de estudo transversal realizado em hospital de ensino. A população do estudo foi composta pelo universo de notificações de queda enviadas para o Núcleo de Segurança do Paciente. A variável dependente foi alto risco para queda. A mensuração do risco de queda foi realizada de acordo com o *Medication Fall Risk Score*. Foram realizadas as análises descritiva, univariada e multivariada.

**Resultados:**

Das 125 notificações de queda incluídas no estudo, 38 (30,4%) foram notificadas em 2014, 26 (20,8%) em 2015 e 61 (48,8%) em 2016. Metade dos pacientes (63; 50,4%) foram classificados na categoria alto risco de queda, e 74 (59,2%) apresentaram dois ou mais fatores de risco para o evento. As classes de medicamentos mais frequentes foram opioides (25%), ansiolíticos (19,7%), betabloqueadores (9,9%), antagonistas de angiotensina II (7%) e bloqueadores de canais de cálcio seletivos com efeitos principais vasculares (7%). Após a análise ajustada, os fatores associados com queda foram amputação (*odds ratio*: 14,17), sexo feminino (*odds ratio*: 2,98) e dor intensa (*odds ratio*: 5,47).

**Conclusão:**

Os medicamentos são importante fator contribuinte para a queda intra-hospitalar, e o *Medication Fall Risk Score* contribui para a identificação de pacientes com alto risco de quedas.

## INTRODUCTION

In-hospital falls are among the most frequent adverse events and contribute to increased morbidity and mortality, longer lengths of stay, and higher health care costs. Moreover, they can affect the quality of life of inpatients.^(^ ^1^ ^)^ In Brazil, in 2014, falls ranked third as adverse events most frequently reported by hospitals.^(^ ^2^ ^)^

The main victims of in-hospital falls are older adults, due to changes inherent to senility and senescence, increased incidence of chronic diseases, and the consequent use of multiple medications. Due to the population aging, falls among the older adults have become a public health problem, which can lead to decreased functionality and increased health care costs. The risk of falling has a greater impact on older age ranges.^(^ ^3^ ^-^ ^5^ ^)^ This may lead to several complications, such as injuries, fractures, traumatic brain injury, and the fear of falling again, which are associated with immobility and loss of independence.^(^ ^3^ ^,^ ^6^ ^-^ ^8^ ^)^

Falls are multifactorial events, and an increased risk of falling can result from physiological changes, frailty and the use of drugs.^(^ ^3^ ^,^ ^9^ ^,^ ^10^ ^)^ Other intrinsic risk factors for in-hospital falls include agitation, dizziness, confusion, muscle weakness, unstable gait, hypovolemia and hypotension. Extrinsic risk factors related with the hospital environment include improper lighting in wards and rooms, oddly located furniture, slippery floors, non-adapted bathrooms, and stairs.^(^ ^1^ ^,^ ^5^ ^,^ ^6^ ^,^ ^9^ ^)^

The Patient Safety Committee (PSC) has the role of adopting strategies to prevent falls inside hospital facilities.^(^ ^11^ ^)^ Important preventive measures include the identification of fall-risk-increasing drugs (FRIDs) used in the facility and the dissemination of information on the risks associated with FRIDs, particularly in the older adults.^(^ ^6^ ^)^

One way of measuring the risk of falls is using the Medication Fall Risk Score (MFRS),^(^ ^12^ ^)^ a validated, easy-to-apply scale. It is recommended by the Agency for Health Care Research and Quality (AHRQ) to be used in in-hospital fall prevention programs, based on the use of medications from drug classes associated with falls. The scale was developed considering the adverse drug reactions profile and their association with falls.^(^ ^12^ ^-^ ^14^ ^)^

Due to the impact of falls on patient safety, it is important to recognize the factors associated with falls and how drugs contribute to their occurrence.^(^ ^15^ ^,^ ^16^ ^)^

## OBJECTIVE

To investigate the use of drugs that increase fall risk among patients with falls reported to the Patient Safety Office of a hospital, and to identify the risk factors associated with a high fall risk.

## METHODS

A retrospective, cross-sectional study of reports of in-hospital falls. The study was conducted at a hospital associated to the Brazilian Unified Health System (SUS) located in the city of Belo Horizonte (State of Minas Gerais), defined as a fully public healthcare organization, responsible for providing services to patients requiring urgent clinical and surgical, trauma and non-trauma care, from a population of 1.1 million inhabitants. The hospital has an installed capacity of 368 inpatient beds and 31 intensive care beds.

The sampling was non-probabilistic and composed of the universe of fall reports submitted to the PSC between January 2014 and December 2016. The PSC of the hospital investigated was founded in 2013 and has a fall prevention subcommittee, established in 2014. Educational activities are developed for the healthcare team, and reporting of fall events is encouraged.

Reports for patients aged under 18 years, falls at home and those relative to patients incorrectly identified in the reporting system were excluded from the study.

Data regarding variables of interest were selected in a Microsoft Excel^®^ worksheet prepared by the PSC. Drug therapy information was completed based on queries to the electronic prescription system of the hospital.

For all patients enrolled in the study, we collected the number of drugs prescribed, the non-drug-related risk factors and the FRIDs administered on the day before the event. The non-drug-related factors collected were those described in the Fall Prevention Protocol of the Ministry of Health.^(^ ^17^ ^)^ They were identified in patient chart notes recorded by physicians, dietitians, physical therapists, occupational therapists and nurses.

Fall-risk-increasing drugs were selected based on the classification developed by Winter et al.,^(^ ^9^ ^)^ and Ek et al.,^(^ ^18^ ^)^ structured according to the anatomical and pharmacological groups of the Anatomical Therapeutic Chemical (ATC) system of the World Health Organization (WHO).^(^ ^19^ ^)^

The fall risk was calculated as per the MFRS scale (Table 1) developed by Beasley et al.^(^ ^12^ ^)^ According to this tool, FRIDs are divided by drug class and may score 1 (low risk), 2 (medium risk) and 3 (high risk). For all patients, we calculated the risk of the event considering the prescription on the day before the fall. If the patient had received more than one drug in each risk category, we multiplied the category score by the number of drugs used. Subjects with score ≥6 were classified as high risk based on the cutoff value suggested by the creators of the MFRS.

**Table 1:** Medication Fall Risk Score

Point value (risk level)	Therapeutic group
3 (high)	Narcotic analgesics, Antipsychotics, Anticonvulsants, Benzodiazepines
2 (medium)	Antihypertensives, cardiac drugs, antiarrhythmics, antidepressants
1 (low)	Diuretics

Source: Beasley B, Patatanian E. Development and implementation of a pharmacy fall prevention program. Hosp Pharm. 2009;44(12):1095-102.^(^ ^12^ ^)^ Score ≥6: high risk of falls.

The dependent variable of the study was a high risk for falls (yes or no), as per the MFRS. Independent variables were related with sociodemographic and psychocognitive factors, health conditions and chronic diseases, functionality, severe obesity, body balance, previous history of fall, and the staff responsible for the patient’s care.

The descriptive analysis was carried out through the preparation of frequency distribution charts for categorical variables and calculation of descriptive statistics for quantitative variables.

When the statistical inference technique required that the variable had a normal distribution, this hypothesis was assessed by the Shapiro-Wilk test (with a significance level set at 5%).

To assess the individual association of each independent qualitative variable with the dependent variable, we used Pearson’s χ^2^ test. Variables with p≤0.20 in Pearson’s χ^2^ test were selected for logistic regression using the Forward Wald method. Variables with p value<0.05 were kept in the final model. The adequacy of the final model was assessed by the Hosmer-Lemeshow test. For statistical analysis, we used the software Statistical Package for Social Science (SPSS) 25.0.

### Ethical aspects

The project was approved by the Institutional Review Board of the hospital under CAAE: 72776217.7.0000.5149, and exempt from informed consent. The organization where the study was performed authorized the investigation as well as access to reports and electronic patient records.

## RESULTS

Of the 148 fall reports to the PSC, 125 were included in the study; in that, 38 (30.4%) were reported in 2014, 26 (20.8%) in 2015, and 61 (48.8%) in 2016. We verified that 92 (73.6%) patients in the reports were male and 68 (54.4%) were aged under 60 years. Mean age was 54.0 years and the standard deviation (SD) was 17.6 years. The occurrence of falls was concentrated on the lower and upper limits of age distribution, with 37 (29.6%) patients under 44 years, 11 (8.8%) between 45 and 49, 11 (8.8) between 50 and 54, 9 (7.2%) between 55 and 59, 25 (20.0%) between 60 and 65, and 32 (25.6) over 65 years. Seventy-three (58.4%) falls reported occurred during the night. We identified that 27 (21.6%) falls resulted in injuries to the patients.

In table 2, we describe the frequency of the non-drug-related risk factors for falls found in the Fall Prevention Protocol of the Ministry of Health. The most frequent risk factors were anemia (40%), age over 65 years (25.6%), difficulty in performing activities of daily living (ADL) (24%), abnormal gait (19.2%), and previous history of fall (18.4%). We observed that 74 (59.2%) patients had two or more non-drug-related risk factors for falls.

**Table 2:** Frequency of risk factors for falls found in the Fall Prevention Protocol of the Ministry of Health described in 125 reports

Variables	n (%)
Sociodemographic characteristics	
Age >65 years	32 (25.6)
Psychocognitive	
Cognitive decline	10 (8.0)
Depression	4 (3.2)
Anxiety	1 (0.8)
Health conditions and chronic diseases	
Stroke	10 (8.0)
Dizziness	2 (1.6)
Seizure	5 (4.0)
Syncope	1 (0.8)
Severe pain	17 (13.6)
Low BMI	12 (9.6)
Anemia	50 (40)
Insomnia	4 (3.2)
Urinary incontinence or urgency	2 (1.6)
Osteoporosis	2 (1.6)
Functionality	
Difficulty in performing ADL	30 (24.0)
Need of ambulatory device	17 (13.6)
Muscle and joint weakness	7 (5.6)
Lower limb amputation	20 (16.0)
Lower limb deformity	1 (0.8)
Sensorial impairment	
Hearing	3 (2.4)
Touch	2 (1.6)
Body balance	
Altered gait	24 (19.2)
Severe obesity	3 (2.4)
Past history of falls	23 (18.4)

BMI: body mass index; ADL: activities of daily living.

In respect to drug therapy, we verified that 74 (59.2%) patients were on 10 or more drugs. The mean risk calculated by the MFRS was 5.5 (SD 3.9), with a minimum of zero and maximum of 18; 63 (50.4%) patients with reported falls were classified by the MFRS into the high fall risk category.

About 84.6% of patients were on FRIDs. In patient prescriptions, we identified 30 different drugs used on the day before the falls, which were classified as FRIDs. The total frequency of the FRIDs used was 284, of which 163 (57.3%) targeted the central nervous system and 121 (42.7%), the cardiovascular system. According to the ATC, level 4, the most frequent drug classes were opioids (25%), anxiolytics (19.7%), beta-blockers (9.9%), angiotensin II antagonists (7%), and vascular-selective calcium-channel blockers (7%) (Table 3).

**Table 3:** Description of drugs used on the day before the falls, classified per anatomical groups and pharmacological subgroups of the Anatomical Therapeutic Chemical (ATC) classification

Código ATC	n (%)
Group N − Nervous System	163 (57.3)
N02A − Opioids: codeine, morphine, tramadol, methadone	71 (25.0)
N05A − Antipsychotics: haloperidol, risperidone, quetiapine	20 (7.0)
N05B − Anxiolytics: alprazolam, clonazepam, diazepam	56 (19.7)
N06A − Antidepressants: amitriptyline, citalopram, fluoxetine, nortriptyline	16 (5.6)
Group C − Cardiovascular System	121 (42.7)
C01D − Vasodilators used in cardiac diseases: isosorbide mononitrate	1 (0.4)
C02A − Antiadrenergic agents, centrally acting: clonidine and methyldopa	2 (0.7)
C02D − Arteriolar smooth muscle, agents acting on: hydralazine	10 (3.5)
C02C − Antiadrenergic agents, peripherally acting: doxazosin	1 (0.4)
C03A – Low-ceiling diuretics: hydrochlorothiazide	7 (2.5)
C03C − High-ceiling diuretics: furosemide	9 (3.2)
C03D − Potassium sparing agents: spironolactone	8 (2.8)
C07A – Beta blocking agents : atenolol, carvedilol, propanolol	28 (9.9)
C08C − Selective calcium channel blockers with mainly vascular effects: amlodipine	20 (7.0)
C08D − Selective calcium channel blockers with direct cardiac effects: diltiazem	1 (0.4)
C09A – Angiotensin converting enzyme inhibitors: captopril, enalapril	14 (4.9)
C09C – Angiotensin II antagonist: losartan	20 (7.0)
Total	284 (100)

The most frequent FRIDs were clonazepam (10.0%) and codeine (10.0%), followed by amlodipine (7.0%), losartan (7.0%), morphine (7.0%), diazepam (7.0%), and tramadol (6.5%).

In the univariate analysis, a high fall risk was associated with the female sex, severe pain, lower limb amputation, and patients seen by the vascular surgery team. After the adjusted analysis by multivariate logistic regression, female subjects had twice as high a chance of being classified as high fall risk when compared to males. Subjects with severe pain had a five-fold higher chance of being classified as high fall risk. Moreover, amputated subjects had a 12-fold higher chance of being classified as high fall risk (Table 4).

**Table 4:** Univariate and multivariate analysis of factors associated to high risk of fall with reported in-hospital fall

Variable	High risk of fall		Univariate analysis		Multivariate analysis	
		
	Yes n (%)	No n (%)	OR (95%CI)*	p value^†^	OR (95%CI)**	p value^†^
Sex						
Female	22 (66.7)	11 (33.3)	2.49 (1.08-5.72)	0.029	2.98 (1.21-7.33)	0.018
Male	41 (44.6)	51 (55.4)	1		1	
Age, years						
≥65	16 (50.0)	16 (50.0)	1	0.958	-	-
<65	47 (50.7)	46 (49.5)	0.98 (0.44-2.19)			
Cognitive decline						
Yes	4 (40.0)	6 (60.0)	1	0.493	-	-
No	59 (51.3)	56 (48.7)	0.63 (0.17-2.36)			
Stroke						
Yes	3 (30.0)	7 (70.0)	1	0.179	-	-
No	60 (52.2)	55 (47.8)	0.39 (0.01-1.60)			
Severe pain						
Yes	14 (82.4)	3 (17.6)	5.61 (1.53-20.68)	0.005	5.47 (1.39-21.51)	0.015
No	49 (45.4)	59 (54.6)	1		1	
Anemia						
Yes	23 (46.0)	27 (54.0)	1	0.422	-	-
No	40 (53.3)	35 (46.7)	0.75 (0.36-1.53)			
Amputation						
Yes	18 (90.0)	2 (10.0)	12.00 (2.65-54.38)	0.00	14.17 (3.01-66.67)	0.001
No	45 (42.9)	60 (57.1)	1		1	
Need of ambulatory device						
Yes	8 (47.1)	9 (52.9)	1	0.767	-	-
No	55 (50.9)	53 (49.1)	0.86 (0.31-2.39)			
BMI						
Yes	7 (58.3)	5 (41.7)	1.43 (0.43-4.76)	0.563	-	-
No	56 (49.6)	57 (45.6)	1			
Past falls						
Yes	11 (47.8)	12 (52.2)	1	0.785	-	-
No	52 (51.0)	50 (49.0)	0.88 (0.36-2.18)			
ADL						
Yes	14 (46.7)	16 (53.3)	1	0.639	-	-
No	49 (51.6)	46 (48.4)	0.82 (0.36-1.87)			
Clinical picture						
Yes	8 (34.8)	15 (65.2)	0.46 (0.18-1.17)	0.097	-	-
No	55 (53.9)	47 (46.1)	1			
Vascular						
Yes	32 (71.1)	13 (28.9)	3.89 (1.77-8.54)	0.001	-	-
No	31 (38.8)	49 (61.3)	1			

* OR (95%CI) estimated by χ^2^ test; ^†^ significant if p<0.05; ^**^ OR (95%CI) estimated by logistic regression method. Verification of adequacy of model: Hosmer-Lemeshow test, χ^2^=1.384; freedom grades =3; p=0.709. OR: odds ratio; 95%CI: 95% confidence interval; BMI: body mass index; ADL: Activities of Daily Life.

## DISCUSSION

The study showed that drugs are an important contributing factor to in-hospital falls, and the MFRS can help identify patients at a high risk for falls. Half of the patients had increased risk for falls according to the MFRS, in addition to having two or more non-drug-related risk factors, which supports that falls are events with multiple determinants. Patients at a high risk for falls in an American study had multiple factors that facilitated falls, which shows the importance of a comprehensive approach, through multifactorial interventions, to reduce patient risk.^(^ ^18^ ^)^

A high fall risk according to the MFRS is related with the use of certain drugs, which can favor the occurrence of falls. The frequent use of FRIDs identified in the case series investigated is in line with a previous descriptive study in an orthopedics department,^(^ ^20^ ^)^ as well as a retrospective study conducted in a Canadian hospital, showing the use of FRIDs was a significant predictor for falls.^(^ ^21^ ^)^

Some drugs markedly contribute to increase the risk of falls, due to certain adverse effects resulting from their use, such as sedation, dizziness, posture disorders which can affect gait and balance, and decreased cognition.^(^ ^7^ ^,^ ^21^ ^)^ Some of these drugs target the central nervous system and others, the cardiovascular system. The findings related with the contribution of these drug classes to the risk of falls are in line with investigations performed in community and hospitalized older adults.^(^ ^21^ ^,^ ^22^ ^)^

Despite other studies showing a higher prevalence of falls among the older adults, our case series showed a higher prevalence among subjects under 44 years, which may be related with the profile of the hospital, which is a reference for urgency and emergency care. Moreover, accidents due to external causes more frequently affect youth and adults.

The use of multiple drugs may contribute to increase prescriptions containing FRIDs, which can increase the incidence of falls. The positive association between polypharmacy (concurrent use of five or more drugs) and fall risk must be better understood, since study results still diverge.^(^ ^23^ ^)^ In the hospital setting, a cutoff point must be established to define the number of drugs in use that contributes to increase the risk of falls.

It is important that pharmacists, along with the multidisciplinary team, employ tools to identify patients at risk, and use strategies to prevent falls. The following strategies are appropriate to the hospital setting: encouraging reporting of falls; reviewing drug therapies; suggesting to prescribers alternative drugs with lower risk; evaluating patients with high fall risk; educating on the safe use of drugs; using non-drug approaches to prevent falls; and communicating the risk of falls associated with certain drugs.^(^ ^12^ ^,^ ^17^ ^)^

Falls can cause injury and, consequently, reduce functionality and prolong patients’ lengths of stay. Moreover, they also contribute to significant increases in public health care spending. The risk of falls with complications increases virtually proportionally with the number of FRIDs, which can often result in hospitalization.^(^ ^23^ ^)^

The number of fall reports dropped from 2014 to 2015, and increased from 2015 to 2016. Promotion of adverse event reporting must be a continuous effort to prevent decrease in number of reports, as seen in 2015. Measures to encourage reporting are important, since, often times, in-hospital falls are neglected by the staff, and also to demystify the culture that reporting leads to blaming and punishment.

After the adjusted analysis, a high fall risk was associated with the female sex, severe pain and lower limb amputation. Women are more predisposed to an increased fall risk,^(^ ^22^ ^,^ ^23^ ^)^ which is attributable to greater frailty when compared to men, higher prevalence of chronic diseases, exposure to household chores and risk activities.^(^ ^22^ ^-^ ^24^ ^)^ Pain also increases the risk of patients falling, since it is associated with mood, mobility and sleep changes.^(^ ^25^ ^)^ Lower limb amputation makes subjects more prone to falling due to difficulty walking and physical disability.^(^ ^25^ ^,^ ^26^ ^)^

Strategies to identify patients at a higher fall risk must be used in the hospital setting to allow for a multiprofessional coordination of efforts aiming to implement interventions for fall prevention. This identification through the use of instruments and a culture of reporting is required for a holistic approach with fall prevention measures. The study hospital, by structuring a reporting program and communicating the fall prevention protocol developed, minding the particularities of the organization, is doing important work towards improving patient safety.

The study employs a comprehensive approach, which looks at fall events in a comprehensive manner, by associating them with both drug-related and non-drug-related factors. However, some limitations should be considered, such as it using a non-probabilistic sample, with predominance of males, and the inclusion of one single reference center, which does now allow for results to be generalized to other patients. Also, this is a retrospective study, which makes it difficult to collect certain data, and this may lead to underestimation of the results. It is worth mentioning that the sample is exclusively composed of subjects with falls reported during the period; the absence of a control group limits understanding of determinants of these adverse events.

Finally, another limitation is the use of the MFRS, which is not validated in Brazil. The scale adopted does not involve subjective and cultural aspects, since it only covers the drug classes identified, using the ATC classification of the WHO. The validation study of the MFRS showed the previously performed reclassification of fall risk – using the Morse Fall Scale, pointed to a discrete improvement in specificity without compromising sensitivity, which confirms the viability of using the Medication Fall Risk Score at hospitals.^(^ ^14^ ^)^ The Morse Fall Scale, although translated and validated in Brazil, requires authorization to be used;^(^ ^17^ ^)^ the MFRS, in turn, is recommended by the Agency for Health Care Research and Quality to be used in in-hospital fall prevention programs.

In terms of future research, we highlight the importance of determining the sensitivity and specificity of the MFRS in Brazilian hospitals, in order to provide a validated scale and contribute to advance investigations of drugs as determinants of falls.

## CONCLUSION

Drugs are important contributors to in-hospital falls, and the Medication Fall Risk Score can help identify patients at a high risk for falls. Falls are multifactorial events determined by drugs and non-drug-related risk factors. Increased fall risk and the presence of two or more non-drug-related risk factors were observed in half of the patients. After the adjusted analysis, the factors associated with a high fall risk were lower limb amputation, female sex and severe pain.
